# Evaluation of Skeletal and Dentoalveolar Changes in Patients With Class II Div 1 Malocclusion Treated With Twin Block Appliance

**DOI:** 10.7759/cureus.49364

**Published:** 2023-11-24

**Authors:** Vaibhav Gandhi, Falguni Mehta, Dolly Patel, Hrishabh Joshi, Aditya Tadinada, Sumit Yadav, Farheen Malek

**Affiliations:** 1 Orthodontics and Dentofacial Orthopaedics, Candian Orthodontic Partners, Red Deer, CAN; 2 Orthodontics and Dentofacial Orthopaedics, Government Dental College and Hospital, Ahmedabad, IND; 3 Orthodontics and Dentofacial Orthopaedics, AMC (Ahmedabad Municipal Corporation) Dental College and Hospital, Ahmedabad, IND; 4 Dentistry, University of Alberta, Edmonton, CAN; 5 Oral and Maxillofacial Radiology, University of Connecticut, Farmington, USA; 6 Growth and Development, University of Nebraska Medical Center, Lincoln, USA; 7 Prosthodontics, Louisiana State University School of Dentistry, New Orleans, USA

**Keywords:** growth modulation, functional therapy, twin block appliance, mandibular retrognathia, class ii malocclusion

## Abstract

Objective

The study was focused on evaluating the change in mandibular morphology following the Twin Block appliance therapy and recording its effect on the maxilla and maxillary dentoalveolar complex. Also, the results of the Twin Block appliance between males and females were compared.

Material and methods

In this two-armed retrospective cephalometric study, 30 patients (mean age 12 years) treated with Twin Block appliance for the period of 8-12 months were chosen, and their records were obtained to analyze. These results were compared with 15 control subjects of the same age group chosen from the American Association of Orthodontics Foundation (AAOF) growth legacy collection: Michigan Growth Study Class II subjects. Cephalometric tracing was done, and data was processed for descriptive statistical analysis.

Results

Paired sample t-test and ANOVA test were performed to evaluate the differences in the pre-treatment (T1) and post-functional (T2) values. ∠ANB showed a mean difference of -4.71°±1.55° for males and 6.22°±6.78° for females, which is significant. The mandibular length (Co-Gn), for male subjects, the mean difference was 5.14±1.74 mm, and for female subjects, it was 6±2mm, which is highly significant; 49.88% of skeletal changes and 50.12% of dentoalveolar changes were reported to bring about Class II correction with Twin Block.

Conclusion

A successful increase in mandibular length was achieved using a Twin Block as a functional appliance. Also, the significant maxillary restraining effect was recorded. More skeletal changes were observed in males than females.

## Introduction

Class II malocclusion comprises a group of specific skeletal, dental, and facial features. The National Health and Nutrition Examination Survey (based on overjet) estimated that approximately 14.7% of the United States population has Class II malocclusion, with prevalence decreasing from 22.6% between eight and 11 years of age to 15.6% between 12 and 17 years of age and then to 13.4% between 18 and 50 years of age [1]. The National Center for Health Statistics reported that 20.4% of 6-11-year-olds have bilateral Class II molar relationships, compared with 14.5% of 12-17-year-olds [2,3]. In India, the prevalence of Class II malocclusion is 14.6% for the age group of 10-13 years, 6% for the age group of 5-9 years, and 3.8% for the age group of 6-14 years [4].

Moyers et al. found the incidence of the normal maxilla in combination with deficient mandible within the Class II population to be approximately 70%, much higher than was formerly recognized [5]. Although maxillary protrusion and mandibular retrognathism are both found to be possible causative factors, McNamara reported that mandibular retrognathism is more common for skeletal Class II malocclusion [6]. McNamara, while studying a group of 277 children with Class II malocclusion, found that 60% of the group had retruded mandibles. Various methods have been introduced to correct this type of malocclusion like functional appliances, extraoral appliances, camouflage treatment, and surgical repositioning [7].

Functional appliances are more commonly used to treat mandibular deficiency in children by stimulating the growth of the mandible. Also, the functional appliances help patients significantly improve the functional relationship of dentofacial structures by eliminating unfavorable developmental factors and improving the muscle environment enveloping the developing dentition. Furthermore, by altering the position of the teeth and supporting tissues, they establish a new and more optimal functional behavioral pattern, which leads to adaptive changes in the bone form and helps the dentofacial complex to achieve its optimal genetic growth potential. A restraining effect on the growth of the maxilla and the maxillary dentoalveolar complex is also reported, along with the stimulation of mandibular growth and mandibular alveolar adaptation with functional appliance treatment.

The most popular myofunctional appliance used for the correction of Class II malocclusion is the “Twin Block” developed by Dr. William J Clark [8]. Twin Blocks are also designed on aesthetic principles to free the patient from the restrictions imposed by a one-piece appliance made to fit in both jaws. It comprises upper and lower blocks at a 70-degree inclination, which frees the mandible from its locked distal position and allows sagittal growth without any hindrance. With the Twin Block appliance, the patient can function quite normally, and thus, it is said to be the most patient-friendly functional appliance to wear. Also, eating and speaking can be accomplished without overly restricting the normal movement of the tongue, lips, and mandible. Nonetheless, one of the added advantages of Twin Block is its versatility of being able to correct transverse discrepancy by incorporating a midline jack screw since deficiency in the transverse plane is often encountered with skeletal class II patients.

Rationale

Many clinical studies have been done on skeletal and dentoalveolar changes associated with functional appliance therapy in treating class II malocclusion, but scientific data is still controversial. Furthermore, no significant demarcation could be found in orthodontic literature regarding applying functional appliances in the growing phase. Thus, the rationale of this study was to expand our knowledge in regard to functional appliance therapy and to focus on quantitative changes in skeletal or dentoalveolar structures associated with it. Furthermore, to the best of our knowledge, this study is the first of its kind focused on overall as well as gender-specific percentage breakdown of skeletal and dentoalveolar effects with Twin Block appliance. 

Specific objective and hypothesis

The aims and objectives of this study were to compare the change in the maxilla and mandible and compare this data with non-treated controls. The study was also focused on evaluating the change in mandibular morphology following the Twin Block appliance therapy and recording its effect on the maxilla and maxillary dentoalveolar complex. Finally, we compared the results of the Twin Block appliance between males and females. The null hypothesis was that there was not any significant difference in terms of mandibular growth between the treatment and control groups.

## Materials and methods

An exemption was obtained from the Institutional Review Board of the Government Dental College and Hospital, Ahmedabad, Gujarat, India, for evaluating lateral cephalometric radiographs archived in the Department of Orthodontics and Dentofacial Orthopaedics. Informed consent was obtained from the parents or guardians of all subjects. This two-armed (experimental and control groups) retrospective study reviewed 30 cephalometric radiographs of patients treated successfully with Twin Block therapy. Study participants were from the age group of 11-14 years, with a mean age of 12. Out of 30 patients, 21 were males and nine were females. As a control, subjects were chosen from the American Association of Orthodontics Foundation (AAOF) growth legacy collection: Michigan growth study class II subjects [9]. We found 15 class II subjects aged 11-13 years. Out of 15 subjects, four were females and 11 were males. The treatment subjects and control samples are from two different sources and thus, it should be considered during the interpretation of the results of this study. The data collection was performed at the Department of Orthodontics and Dentafacial Orthopaedics at the Government Dental College and Hospital, Ahmedabad, India.

Inclusion criteria for the treatment group were class II skeletal malocclusion with orthognathic maxilla and retrognathic mandible, potential growth still left (CVMI stage II/III), full cusp Class II molar relationship with division 1 pattern, positive visual treatment objective (VTO), ANB angle more than 4 degrees, average to horizontal growth pattern, and overjet more than 7mm. The exclusion criteria were set as follows: (i) cases with congenitally missing teeth, (ii) Class I or Class III malocclusion, (iii) VTO negative, (iv) Patients with supernumerary teeth, enlarged/cystic follicle or any other pathology, (v) Orthodontic treatment without the use of Twin Block, (vi) Extraction therapy, (vii) Non-growing patients, (viii) Systemic disease affecting bone of the patients, (ix) cases that had an extraction of teeth for orthodontic purposes, and (x) patients having periodontal disease, orthognathic surgery, or any genetic syndromes. All lateral cephalogram images were de-identified for protected health information (PHI) by authorized personnel before they were used as a part of the study subject. All the subjects in the experimental group were from Asian and particularly South Asian ethnic groups.

After obtaining complete records, including relevant radiographs, models, and photographs, these patients were treated with Twin Block appliance for 8-12 months with an average of 10 months, and post-functional records were obtained.

**Figure 1:**
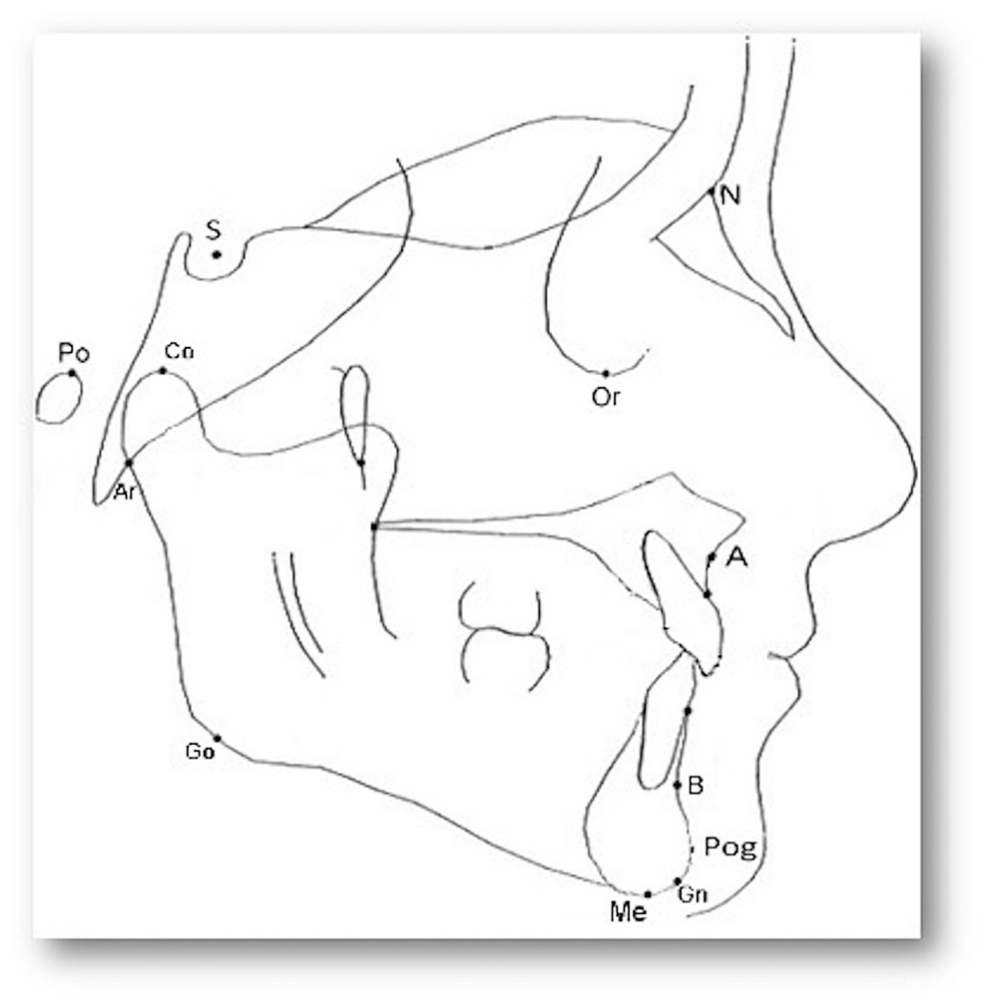
Various landmarks used in the study. Image credit: Authors

**Figure 2:**
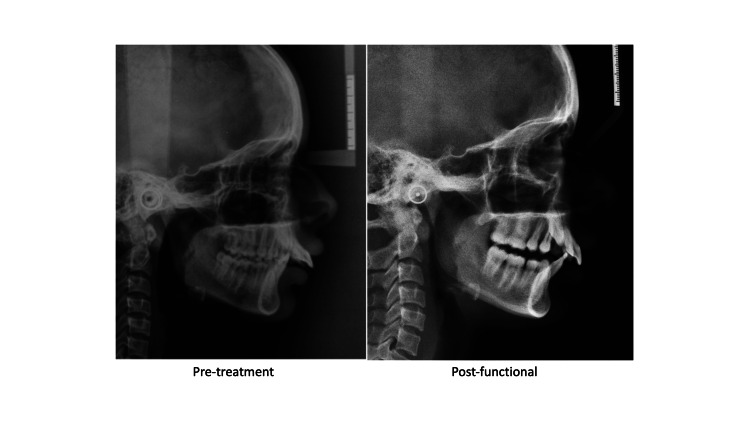
Sample images of pretreatment and post-functional lateral cephalogram of a study participant.

All the radiographs were traced on a standard matte acetate tracing paper in random order by a single operator and repeated at four-week intervals to reduce bias. The investigator reviewed the lateral cephalograms on a light-emitting diode (LED) lightbox under standardized conditions of ambient light and sound. Cephalometric analysis was done on pre-treatment and post-functional cephalogram and non-treated controls from the American Association Of Orthodontists Foundation (AAOF) growth legacy collection. Parameters used were as follows: SNA, SNB, ANB, nasion perpendicular to point A, nasion perpendicular to point Pg, Co-point A, Co-Gn, saddle angle, articular angle, gonial angle (upper and lower), maxillary molar position, mandibular molar position, maxillary incisor position, mandibular incisor position, incisor mandibular plane angle (IMPA), overjet, and overbite (Figures 1, 2). The resultant data was subjected to descriptive statistical analysis. 

Paired sample t-test was performed to evaluate the differences in the pre-treatment (T1) and post-functional (T2) values. Additionally, method error, mode of variance, and reliability of each cephalometric parameter were evaluated. Statistical analyses were computed using GraphPad software (Dotmatics, Boston, Massachusetts).

## Results

Descriptive statistics, including mean difference, standard deviation, and standard error of the mean and p-value of paired sample t-test for male patients from the treatment and control groups are reported in Tables 1, 2, respectively. Eight of the 12 skeletal and four of the seven dentoalveolar parameters showed significant and favorable results with Twin Block appliances for the male treatment group. SNB angle increased by 3.86±1.31 degrees, and the ANB angle decreased by 4.71±1.55, which were statistically significant (p<0.0001). Mandibular length (Co-Gn) showed a significant increase of 5.14±1.74 mm (p<0.0001). The lower gonial angle increased by 3.24±2.55 (<0.0001). Overjet (-6.64±2.40 mm) and Overbite (-3.81±2.06 mm) showed significant reduction (p<0.0001) after treatment with Twin Block (Table 1). On the other hand, the control group showed a 0.91±1.04 degree change in SNB angle (p=0.16) and -0.73±1.35 degree change in ANB angle (p=0.1), and the difference was not statistically significant. There was a 2.09±2.55 mm change in the Co-Gn distance (p=0.02). Overjet and overbite did not show a significant change in the male control group (Table 2).

**Table 1:** Mean difference, standard deviation, and p-value of all pre-treatment and post-functional parameters for the male treatment group. P >0.05; not significant.

Serial No.	Parameter	Mean Difference ± SD	Standard error	p-value
Skeletal				
1	SNA angle	(-0.67)±1.15	0.25	0.02
2	SNB angle	3.86±1.31	0.29	<0.001
3	ANB angle	(-4.71)±1.55	0.34	<0.001
4	Nasion ⊥ to point A	(-0.14)±1.28	0.28	<0.001
5	Nasion ⊥ to point Pg	5.81±3.22	0.7	<0.001
6	Co-point A length	(-0.62)±2.04	0.44	0.18
7	Co-Gn length	5.14±1.74	0.38	<0.001
8	Saddle angle (N-S-Ar)	(-0.29)±1.19	0.26	0.28
9	Articular angle ( S-Ar-Go)	(-2.86)±2.13	0.46	<0.001
10	Gonial Angle (Ar-Go-Gn)	3.33±3.62	0.79	<0.001
11	Upper Gonial Angle (Ar-Go-N)	0.10±4.47	0.98	0.92
12	Lower Gonial Angle (N-Go-Gn)	3.24±2.55	0.56	<0.001
Dental				
1	Maxillary molar positiomn	(-0.67)±0.66	0.14	<0.001
2	Mandibular molar position	5.76±1.18	0.26	0
3	Maxillary incisor position	(-1.12)±1.80	0.39	0.01
4	Mandibular incisor position	0.57±1.43	0.31	0.08
5	IMPA angle	0.81±2.69	0.59	0.18
6	Overjet	(-6.64)±2.40	0.52	<0.001
7	Overbite	(-3.81)±2.06	0.45	<0.001
P >0.05; not significant.				

**Table 2:** Mean difference, standard deviation, and p-value of all pre-treatment and post-functional parameters for the male control group. P>0.05; not significant. IMPA: incisor mandibular plane angle

Serial No.	Parameter	Mean Difference ± SD	Standard error	p-value
Skeletal				
1	SNA angle	0.18±1.47	0.44	0.69
2	SNB angle	0.91±1.04	0.31	0.16
3	ANB angle	(-0.73)±1.35	0.41	0.1
4	Nasion ⊥ to point A	(-0.55)±1.69	0.51	0.31
5	Nasion ⊥ to point Pg	0.55±2.21	0.67	0.43
6	Co-point A length	0.73±2.37	0.71	0.33
7	Co-Gn length	2.09±2.55	0.77	0.02
8	Saddle angle (N-S-Ar)	(-1.82)±4.56	1.37	0.22
9	Articular angle ( S-Ar-Go)	1.64±5.35	1.61	0.34
10	Gonial Angle (Ar-Go-Gn)	(-1.18)±2.23	0.67	0.11
11	Upper Gonial Angle (Ar-Go-N)	1.00±0.82	0.41	0.09
12	Lower Gonial Angle (N-Go-Gn)	(-0.18)±1.66	0.5	0.72
Dental				
1	Maxillary molar positiomn	0.27±1.01	0.3	0.39
2	Mandibular molar position	0.64±2.06	0.62	0.33
3	Maxillary incisor position	(-0.27)±1.35	0.41	0.52
4	Mandibular incisor position	0.64±1.96	0.59	0.31
5	IMPA angle	1.36±1.03	0.31	0
6	Overjet	(-0.45)±1.57	0.47	0.36
7	Overbite	(-0.27)±1.01	0.3	0.39

Descriptive statistics, including mean difference, standard deviation, and standard error of the mean and p-value of paired sample t-test for female patients from the treatment and control groups, are reported in Tables 3, 4, respectively. Four of the 12 skeletal and four of the seven dentoalveolar parameters showed significant and favorable results with Twin Block appliances for the female treatment group. SNB angle increased by 2.78±1.30 degrees, and the nasion ⊥ to point Pg angle increased by 4.22±0.67, statistically significant (p<0.0001). Mandibular length (Co-Gn) showed a significant increase of 6.00±2.00 mm (p<0.0001). Overjet (-4.78±1.64 mm) and overbite (-2.11±1.45 mm) showed significant reduction (p<0.0001) after treatment with Twin Block (Table 3). On the other hand, the control group showed a 0.25±0.50 degree change in SNB angle (p=0.39) and a 0-degree change in ANB angle, and the difference was not statistically significant. There was a 3±4.08 mm change in the Co-Gn distance (p=0.24). Overjet and overbite did not show a significant change in the female control group (Table 4).

**Table 3:** Mean difference, standard deviation, and p-value of all pre-treatment and post-functional parameters for the female treatment group. P>0.05; not significant.

Serial No.	Parameter	Mean Difference ± SD	Standard error	p-value
Skeletal				
1	SNA angle	(-1.11)±0.93	0.31	0.01
2	SNB angle	2.78±1.30	0.43	<0.001
3	ANB angle	(-6.22)±6.78	2.26	0.03
4	Nasion ⊥ to point A	(-0.89)±1.05	0.35	0.04
5	Nasion ⊥ to point Pg	4.22±0.67	0.22	<0.001
6	Co-point A length	0.33±1.66	0.55	0.56
7	Co-Gn length	6.00±2.00	0.67	<0.001
8	Saddle angle (N-S-Ar)	(-0.56)±1.33	0.44	0.25
9	Articular angle ( S-Ar-Go)	(-2.78)±1.20	0.4	<0.001
10	Gonial angle (Ar-Go-Gn)	2.22±2.28	0.76	0.02
11	Upper gonial angle (Ar-Go-N)	(-0.22)±2.68	0.89	0.81
12	Lower gonial angle (N-Go-Gn)	2.11±2.57	0.86	0.04
Dental				
1	Maxillary molar positiomn	(-1.33)±1.00	0.33	<0.001
2	Mandibular molar position	4.33±1.00	0.33	<0.001
3	Maxillary incisor position	(-1.44)±2.55	0.85	0.13
4	Mandibular incisor position	(-0.56)±1.74	0.58	0.37
5	IMPA angle	(-0.11)±2.32	0.77	0.89
6	Overjet	(-4.78)±1.64	0.55	<0.001
7	Overbite	(-2.11)±1.45	0.48	0

**Table 4:** Mean difference, standard deviation, and p-value of all pre-treatment and post-functional parameters for the female control group. P>0.05; not significant. IMPA: incisor mandibular plane angle

Serial No.	Parameter	Mean Difference ± SD	Standard error	p-value
Skeletal				
1	SNA angle	0.25±0.50	0.25	0.39
2	SNB angle	0.25±0.50	0.25	0.39
3	ANB angle	0	0	NP
4	Nasion ⊥ to point A	0.25±0.50	0.25	0.39
5	Nasion ⊥ to point Pg	0.25±1.26	0.63	0.72
6	Co-point A length	0±3.74	1.87	1
7	Co-Gn length	3±4.08	2.04	0.24
8	Saddle angle (N-S-Ar)	1.25±1.71	0.85	0.24
9	Articular angle ( S-Ar-Go)	(-1.75)±4.19	2.1	0.47
10	Gonial angle (Ar-Go-Gn)	0.50±1.29	0.65	0.5
11	Upper gonial angle (Ar-Go-N)	1.00±0.82	0.41	0.09
12	Lower gonial angle (N-Go-Gn)	(-0.50)±1.00	0.5	0.39
Dental				
1	Maxillary molar position	0.5±1.91	0.96	0.64
2	Mandibular molar position	1.00±1.41	0.71	0.25
3	Maxillary incisor position	0.75±0.50	0.25	0.06
4	Mandibular incisor position	0.50±1.29	0.65	0.5
5	IMPA angle	1.00±2.83	1.41	0.53
6	Overjet	0.50±1.00	0.5	0.39
7	Overbite	0	0	NP

Independent sample T-test was performed to compare the parameters between treatment and control groups. Out of 12 skeletal parameters, eight showed a significant difference in comparison of treatment vs. control groups. There was a significant difference for SNB (2.80), ANB (-4.630), nasion ⊥ to point Pg (4.870), Co-Gn (3.27 mm), articular angle (-3.570), gonial angle (3.730), and lower gonial angle (3.170). Four dental parameters showed significant differences between treatment and control groups, including overjet (-5.88 mm) and overbite (-3.1 mm) (Table 5).

**Table 5:** Independent sample T-test between treatment and control groups. P>0.05; not significant. IMPA: incisor mandibular plane angle

Serial No.	Parameter	Group	Mean ± SD	Standard error	Mean difference	p-value
1	SNA angle	Treatment	(-0.80) ± 1.1	0.2	-1	0.009
		control	0.20±1.26	0.33		
2	SNB angle	Treatment	3.53±1.38	0.25	2.8	<0.001
		control	0.73±0.96	0.25		
3	ANB angle	Treatment	(-5.17)±3.85	0.7	-4.63	<0.001
		control	(-0.53)±1.19	0.31		
4	Nasion ⊥ to point A	Treatment	(-0.37)±1.25	0.23	-0.03	0.937
		control	(-0.33)±1.5	0.39		
5	Nasion ⊥ to point Pg	Treatment	5.33±2.8	0.51	4.87	<0.001
		control	0.47±1.96	0.51		
6	Co-Point A length	Treatment	(-0.33)±1.95	0.36	-0.87	0.222
		control	0.53±2.67	0.69		
7	Co-Gn length	Treatment	5.40±1.83	0.33	3.07	<0.001
		control	2.33±2.89	0.75		
8	Saddle angle (N-S-Ar)	Treatment	(-0.37)±1.22	0.22	0.63	0.442
		control	(-1.00)±4.17	1.08		
9	Articular angle (S-Ar-Go)	Treatment	(-2.83)±1.88	0.34	-3.57	0.001
		control	0.73±5.16	1.33		
10	Gonial angle (Ar-Go-Gn)	Treatment	3.00±3.28	0.6	3.73	<0.001
		control	(-0.73)±2.12	0.55		
11	Upper Gonial angle (Ar-Go-Na)	Treatment	0.00±3.97	0.73	0.47	0.666
		control	(-0.47)±1.64	0.42		
12	Lower Gonial angle (Na-Go-Gn)	Treatment	2.90±2.56	0.47	3.17	<0.001
		control	(-0.27)±1.48	0.38		
Dental						
Serial No.	Parameter	Group	Mean ± SD	Standard error	Mean difference	p- value
1	Maxillary molar position	Treatment	(-0.87)±0.82	0.15	-1.2	<0.001
		control	0.33±1.23	0.32		
2	Mandibular molar position	Treatment	5.33±1.30	0.24	4.6	<0.001
		control	0.73±1.87	0.48		
3	Maxillary incisor position	Treatment	(-1.22)±2.02	0.37	-1.22	0.039
		control	0.00±1.25	0.32		
4	Mandibular incisor position	Treatment	0.23±1.59	0.29	-0.37	0.486
		control	0.60±1.76	0.46		
5	IMPA angle	Treatment	0.53±2.58	0.47	-0.73	0.32
		control	1.27±1.58	0.41		
6	Overjet	Treatment	(-6.08)±2.34	0.43	-5.88	<0.001
		control	(-0.20)±1.47	0.38		
7	Overbite	Treatment	(-3.30)±2.04	0.37	-3.1	<0.001
		control	(-0.20)±0.86	0.22		

Analysis was conducted to evaluate the percentage of changes between skeletal and dental changes due to treatment with Twin Block appliance. For the combined (male and female) subjects, 49.88% of changes were skeletal, and 50.12% were due to dental effects. In male subjects, 57.80% were due to skeletal and 42.20% changes were due to dental effects, whereas 36.30% changes were due to skeletal effects and 63.70% were due to dental effects in female subjects (Table 6).

**Table 6:** Mean, standard deviation, standard error, mean difference, p-value, and comparative percentage between skeletal and dental parameters for overall, male, and female treatment groups.. P>0.05; not significant.

Parameter	N	Mean ± SD	Standard error	Mean difference	p-value	Percentage
Overall						
Skeletal	360 = (30x12)	3.00±2.67	0.141	-0.01	0.949	49.88%
Dental	210 = (30x07)	3.01±2.39	0.165			50.12%
Male						
Skeletal	252 = (21x12)	1.00±3.91	0.247	1.73	<0.0001	57.80%
Dental	147 = (21x07)	(-0.73)±4.05	0.334			42.20%
Female						
Skeletal	108 = (09x12)	0.49±3.97	0.382	1.35	0.021	36.30%
Dental	063 = (09x07)	(-0.86)±3.06	0.385			63.70%

The inter-reliability of the skeletal and dental parameters was between 0.88 and 0.98 (Table 7).

**Table 7:** The reliability of the measurement of various cephalometric variables. IMPA: incisor mandibular plane angle

Parameter	Method error	Mode of variance	Reliability
SNA	0.25	1.53	0.9
SNB	0.67	3.34	0.98
ANB	0.44	15.1	0.93
Nasion to point A	0.62	1.73	0.97
Nasion to Pg	0.61	12.03	0.94
Co-point A	0.26	4.95	0.96
Co-Gn	0.65	7.01	0.98
Saddle angle (N-S-Ar)	0.28	6.61	0.92
Articular angle (S-Ar-Go)	0.25	13.69	0.88
Gonial angle (Ar-Go-Gn)	0.66	11.69	0.99
Upper gonial angle (Ar-Go-Na)	0.72	11.32	0.88
Lower gonial angle (Na-Go-Gn)	0.6	7.32	0.89
maxillary molar position	0.59	1.25	0.92
mandibular molar position	0.36	7.03	0.99
maxillary incisor position	0.38	3.51	0.88
mandibular incisor position	0.62	2.69	0.97
IMPA	0.31	5.31	0.9
Overjet	0.67	12.17	0.88
Overbite	0.69	5.15	0.92

## Discussion

SNA angle showed little reduction post functionally from 83° to 82.33° (-0.67° ± 1.15°) for the male treatment group and from 81.67° to 80.56° (-1.11° ± 0.93°) for females, and both are significant. Along with SNA, N perpendicular to point A for female subjects showed significant readings (-0.33 to -1.22 mm, - 0.89±1.05 mm). However, for male subjects and the control group, this parameter showed non-significant results. These findings suggest that maxillary forward growth had been restrained and produced a headgear effect (Tables 1, 3). A similar restraining effect of the maxilla can be seen in studies by Tulloch et al. [10] and Keeling et al. [11], who studied the effect of Bionator, and Trenouth [12], and Obrien et al. [13], who studied the effect of Twin Block.

ANB angle decreased for the treatment group, depicting improvement in the anteroposterior relationship of the maxilla and mandible. For male subjects, the mean difference was -4.71°±1.55° and for female subjects, it was -6.22°±6.78° which was significant. The correction of ANB angle was mainly due to an increase in SNB angle. For male subjects, the mean difference was 3.86°±1.31° and for female subjects, it was 2.78°±1.30°, along with a small reduction in SNA angle (Tables 1, 3). An increase in SNB angle due to an increase in mandibular length and restriction of maxillary growth in the sagittal plane was likely to improve ANB angle. This increase in SNB angle was highly significant (p≤0.001). Similar results in favor of the Twin Block appliance were reported by Llling et al. [14], who compared Bass, Bionator, and Twin Block appliances. They concluded that the Twin Block and, to a lesser extent, Bionator produced the most effective sagittal and vertical changes in the Class II malocclusion in mandibular retrognathic faces. Furthermore, the mandibular length (Co-Gn) increased for male subjects; the mean difference was 5.14±1.74mm, and 6±2mm was for female subjects, which was significant (p ≤ 0.001), supporting the above data. The distance from nasion perpendicular to the pogonion point (N perpendicular to point Pg) was decreased. For male subjects, the mean difference was -5.81±3.22mm, and for females, it was -4.22±0.67mm, which was reported as significant (p ≤ 0.001) (Tables 1, 3). These results indicate that changes in a maxillomandibular relationship were due to changes in the position of the mandible (Tables 1, 3). ANB, SNB, and N perpendicular to point Pg showed non-significant results for control male and female subjects (Tables 2, 4). Similar results have been reported by Šidlauskas [15], Lund and Sandler [16], Toth and McNamara [17], Mills [18], Trenouth [12], Llling et al. [14], and Jena et al. [19].

Saddle angle (N-S-Ar), which signifies the position of the mandible with respect to the cranial base, showed non-significant results for both treatment and control subjects. However, the articular angle (S-Ar-Go) showed a decrease of -2.86° ± 2.13° for the male treatment group and -2.78±1.20 for the female treatment group, which was significant. A decrease in this angle suggests forward repositioning of the mandible, causing an opening of the bite, which is clinically relevant in correcting deep bite. For the control group, both male and female subjects showed non-significant results for saddle angle (Tables 1-4).

Gonial angle (Ar-Go-Gn) showed an increase of 3.33° ± 3.62° for the male treatment group and 2.22°±2.28° for the female, which was highly significant (p≤0.001). However, the upper gonial angle (Ar-Go-N) showed non-significant readings, whereas the lower gonial angle (N-Go-Gn) was increased by 3.24° ± 2.55° for the male treatment group and 2.11°±2.57° for females, which counted as significant. An increase in the lower gonial angle has an effect on the increase in the mandibular plane angle. These findings are in accordance with Pancherz [20], who found an increase in the gonial angle of the patients treated with the Herbst appliance. He determined that by changing the muscle functions or by sagittal directing condylar growth, there could be some resorption on the gonial region. This growth modification, as suggested by the increase in gonial angle, has previously been described as "posterior mandibular morphogenetic rotation," a biological mechanism leading to greater increments in total mandibular length and, thus, efficiently improving the skeletal sagittal relationships in Class II malocclusion [21,22]. All three, gonial angle, upper and lower gonial angle, showed non-significant readings for control groups (Tables 1-4).

Maxillary molar showed distal movement of -0.67±0.66mm for the male treatment group and -1.33±1mm for females, which was significant (p≤0.001). These values indicate that the Twin Block appliance shows a headgear effect on the upper molars. Maxillary incisors also showed distal movement from -1.12±1.80 mm for the male treatment group and -1.44±2.55 mm for females (Tables 1 and 3). This is probably due to the placement of a labial bow in the upper plate of the Twin Block, a modification incorporated in the Twin Block Appliance. This is in contrast to a study by Yaqoob et al., who observed no changes in pre and post-functional cephalograms of the patients treated with Twin Block with and without a labial bow [23]. Both maxillary molar and incisor positions for the control group showed non-significant results (Tables 2, 4).

Mandibular molar showed mesial movement of 5.76±1.18mm and 4.33±1.00 mm for the male and female treatment groups, which was highly significant (p≤0.001), along with an increase in mandibular length (Co-Gn) at the end of the post functional phase (Tables 1, 3). This is in agreement with Trenouth’s readings, which say that Twin Block not only results in forward positioning of the mandible but also lengthens it, as shown by linear measurements [12]. Mandibular incisor position (1 to NB linear) and IMPA showed non-significant flaring for the treatment group (Tables 1 and 3). This result is also supported by Trenouth, who reported a similar restraining effect on lower anteriors (1.4°) using a labial bow on the lower anteriors [12]. In the current study, islet clasps on lower anterior teeth had likely maintained a lower incisor position. Similar results with lower incisors were shown in the studies carried out by Toth and Mcnamara (2.8°) [17] and Šidlauskas (3.2°) [15]. In contrast to these studies, Lund and Sandler [16] and Mills and Mcculloch [18], in their research, found significantly lower incisor proclination during treatment with Twin Block.

Overjet decreased significantly -6.64±2.40 mm for the male treatment group and -4.78±1.64 mm for the female following the Twin Block therapy. The change was statistically significant (p≤0.001) (Tables 1, 3). This effect was mainly due to the forward growth of the mandible with less lingual tipping of the upper anteriors. This is in contrast with the study by Antanas Šidlauskas [15] and Eden Y. Lau et al. [24], who reported lingual tipping of the upper incisors, thereby reducing overjet, whereas in this study, the overjet reduction was mainly due to forward growth of the mandible. Overbite decreased significantly to 3.81±2.06 mm for the male treatment group and 2.11±1.45mm for females (p≤0.001)(Tables 1 and 3). This might be due to a decrease in the articular angle and an increase in the lower gonial angle, thereby modifying the direction of growth of the mandible. Similar results were obtained in the study by Antanas Sidlauskas [15].

For the Twin Block appliance, SNA, SNB, ANB, N perpendicular to Pg, Co-Gn, articular angle, lower gonial angle, mandibular molar position, maxillary molar position, overjet, and overbite all showed significant differences (Table 5). This proves that the Twin Block appliance is efficient inducing change in mandibular growth in growing subjects.

Furthermore, in the current study, 49.88% of skeletal changes were found along with 50.12% of dentoalveolar changes to bring about Class II correction with Twin Block Appliance. This indicates that with Twin Block treatment, 49.88% of 6.08 mm ± 2.34 mm overjet correction was achieved by skeletal changes, and the rest, 50.12%, by dentoalveolar changes (Table 6). This result is supported by the study of Sidlauskas [15], which indicates that with Twin-Block treatment, about 40% of 4.7 mm net overjet correction was achieved by skeletal changes (0.3 mm maxillary growth restriction and 1.7 mm mandibular base increase), and the rest, almost 60% - by dentoalveolar changes (1.3 mm lower incisors advancement and 1.4 mm upper incisors backward movement).

Additionally, males showed 57.8% of skeletal change and 42.2% of dentoalveolar change. Females showed 36.3% of skeletal change and 63.7% of dentoalveolar change with the Twin Block appliance (Table 6). This is in agreement with the study of Heather et al. [14] and Mammandra et al. [25], where more skeletal correction was observed in males than females.

Twin Block, being a versatile appliance, can be modified effectively as per requirements by the addition of expansion screws, labial bows, islets, headgear, and concord face bows. It is a widely used functional appliance for correction of Class II malocclusion with retrognathic mandible. This appliance changes the size and position of the mandible, as evidenced by changes in articular angle, Co-Go-Gn angle, SNB angle, and N perpendicular to point Pg. It can also cause a change in the direction of growth of the mandible, which is evident from the change in lower gonial angle and overbite. Class I molar relationship was achieved partly due to mandibular growth and partly by mesial movement of the mandibular first molar with slight distal movement of the maxillary first molar. Maxillary forward growth was restrained, and the mandibular apical base moved forward in relation to the cranial base, which proved that Twin Block produces a headgear effect with an increase in mandibular length, thereby achieving Class I occlusion and improving the profile.

Similar to any other research project, this study has several limitations. This was a retrospective study, and due to ethical concerns, we could not enroll untreated class II patients from the same geographic area. Also, we did not consider the cervical vertebral maturity indicator (CVMI), hand-wrist radiographs, or middle phalanx of the middle finger (MP3) as skeletal maturity indicators and correlate them with the skeletal or dental correction with Twin Block appliance. It will be interesting to explore the results of any long-term prospective randomized controlled trial that can solve the controversy on the efficiency of functional appliances. 

## Conclusions

The Twin Block appliance achieved a significant increase in mandibular length. A significant decrease in articular angle with an increase in lower gonial angle showed forward and downward positioning of the mandible. This also caused a significant decrease in overjet and overbite. Even though it was to a lesser extent, there was a significant maxillary restraining effect on the maxilla, which enhanced the effect of Twin Block. More skeletal changes are observed in males than females. However, further investigation with a larger sample size is recommended to strengthen these results. Class II molar relationship was corrected by mandibular sagittal growth and distal movement of the upper molar to Class I, which is an efficient factor in counteracting occlusal relapse.

Twin Block appliance brings about the correction of Class II malocclusion by both skeletal and dentoalveolar changes. The clinical consequence is that active treatment of skeletal disharmony with functional appliance can be followed by the phase of fixed appliance therapy to refine occlusion and give stability to the newly established intermaxillary relationship.
